# Knowledge and perceptions of medical practitioners on the role of nutrition in systemic sclerosis

**DOI:** 10.1136/jsrd-2026-000005

**Published:** 2026-05-26

**Authors:** Elisavet Efthymiou, Eleni C Pardali, Arriana Gkouvi, Alexandros Mitropoulos, Christina G Katsiari, Eirini Koidou, Dimitrios G Goulis, Markos Klonizakis, Dimitrios P Bogdanos, Maria G Grammatikopoulou

**Affiliations:** 1 Immunonutrition Unit, Department of Rheumatology & Clinical Immunology, Medical School, University of Thessaly, Larissa, Greece; 2 Lifestyle, Exercise and Nutrition Improvement (LENI) Research Group, Department of Nursing and Midwifery, College of Health, Wellbeing and Life Sciences, Sheffield Hallam University, Sheffield, UK; 3 Department of Rheumatology and Clinical Immunology, Medical School, University of Thessaly, Larissa, Greece; 4 Department of Human Performance, Faculty of Physical Education and Sports Sciences, Aristotle University of Thessaloniki, Serres, Greece; 5 Unit of Reproductive Endocrinology, 1st Department of Obstetrics and Gynaecology, Medical School, Aristotle University of Thessaloniki, Thessaloniki, Greece

**Keywords:** Lifestyle Medicine, Diet, Medical Education, Dysphagia, Scleroderma, Systemic

## Abstract

**Objectives:**

Systemic sclerosis (SSc) presents complex manifestations that impact disease progression and quality of life. Despite the burden of gastrointestinal (GI) and nutrition-related symptoms, a lack of consensus is apparent regarding the appropriate dietary strategies to support SSc management. The present study aimed to explore the perspectives of rheumatologists regarding the importance of nutrition counselling and nutrition-related patients’ needs.

**Methods:**

A cross-sectional study was conducted using a sample of 74 rheumatologists in Greece. The Views on Education and Nutrition in Scleroderma (VENUS) questionnaire was employed. The tool assesses three domains: demographics, scleroderma management and nutrition education approaches. Descriptive statistics summarised the findings, while comparisons and correlations were performed, where appropriate.

**Results:**

Physicians noted that patients with SSc requested dietary advice for symptom control (58.1%), improving general nutrition knowledge (32.4%) and adhering to specialised diets (29.7%). While practitioners recognised the importance of nutrition in SSc care, they emphasised the value of structured support, including face-to-face consultations (51.4%) and the integration of dietitians into multidisciplinary care teams (40.5%). Patients frequently sought nutritional advice, especially regarding reflux management (74.3%), soft-food diet (47.0%) and less commonly other dietary regimens. Regarding symptomatology, most rheumatologists reported pain, digital ulcers and GI discomfort in over half of their patients. Most commonly reported GI symptoms included reflux (89.2%), bloating (55.4%), heartburn (54.1%) and dysphagia (52.7%).

**Conclusion:**

Patients with SSc experience significant pain and GI disturbances and actively seek dietary guidance. Rheumatologists acknowledge the importance of nutrition in disease management and highlight the need for comprehensive multidisciplinary care models involving dietitians, aiming to improve patient outcomes.

## Introduction

 Systemic sclerosis (SSc) is a systemic autoimmune rheumatic disease that continues to pose significant challenges for clinicians, while being associated with high mortality rates.^1^ It is characterised by fibrosis of the skin and lungs, with potential involvement of other internal organs.^1^ Commonly observed manifestations include digital ulcers, inflammation, fatigue, progressive cardiopulmonary disease and gastrointestinal (GI) dysfunction.^1^ Approximately 17.6 individuals per 100 000 people are affected by SSc, classifying it as a rare disease^2^; however, SSc symptomatology and often delayed diagnosis has a grave effect on patients’ quality of life (QoL).^3^

GI involvement has been observed in up to 90% of patients with SSc and represents one of the major contributors to morbidity and mortality.^4^ Fibrosis, a term denoting scarring of the tissue, can affect both the upper and lower GI tract, leading to a wide range of symptoms. Upper GI manifestations include dysphagia, gastro-oesophageal reflux disease and gastroparesis, while lower GI involvement may cause diarrhoea, constipation and intestinal pseudo-obstruction.^5^ These complications have been shown to yield a significant impact on patients’ ability to eat, digest and absorb nutrients, contributing to malnutrition,^6^ sarcopenia^7^ and reduced QoL.^8^

Managing SSc places a significant strain on healthcare resources due to increased patient needs and expenses. Individualised nutritional therapy may serve as a critical element of comprehensive care.^9^ The European League Against Rheumatism and the British Society for Rheumatology have both emphasised the critical role of nutrition in SSc management.^10 11^ In addition to that, a North American expert panel suggested incorporating malnutrition screening in SSc, although this has not yet been implemented in routine clinical practice.^12^ Nevertheless, clinicians frequently report lack of confidence and knowledge in providing evidence-based nutritional guidance.^13^ On the other hand, patients with SSc actively seek information regarding nutrition and dietary strategies to assist in the management of symptoms. Of note, according to research,^14^ patients emphasise the necessity of personalised nutrition counselling as an integral part of comprehensive SSc care. A study conducted in Australian rheumatologists acknowledged the frequency of GI symptoms and the importance of providing nutritional advice to the patients.^15^ Therefore, the present study aimed to determine the perspectives of rheumatologists in Greece regarding nutrition and dietary counselling in SSc.

## Materials and methods

### Sample recruitment and characteristics

Practicing rheumatologists and rheumatology residents were recruited online (from October 2024 to May 2025), contacted through email or scientific associations or recruited through rheumatology-related congresses. All registered members of the society were invited to participate in the study. A total of 75 responses were collected; however, the final sample consisted of 74 participants, since one participant did not provide consent. Table 1 presents the demographic and general characteristics of the sample.

**Table 1:** Characteristics of our sample (n=74)

Variables	Characteristics	n (%)
Sex	Men/women	41 (55.4%)/33 (44.6%)
Educational level	MD/MSc/PhD/other degree	23 (31.1%)/15 (20.3%)/34 (45.9%)/2 (2.7%)
Medical practice site	Private practice/Private hospital/Public hospital/University hospital/Military hospital/not specified	36 (48.6%)/2 (2.7%)/18 (24.3%)/16 (21.6%)/1 (1.4%)/1 (1.4%)
Practice region	Thessaly/Central Greece/Macedonia/Epirus/Peloponnese/Islands/other	27 (36.5%)/18 (24.3%)/14 (18.9%)/1 (1.4%)/5 (6.8%)/5 (8.2%)/3 (4.1%)
Time spent in scleroderma clinic each week	≥ 5 days/3–4 days/1–2 days/<1 day	11 (14.9%)/13 (17.6%)/19 (25.7%)/31 (41.9%)

MD, Medical doctor; MSc, Master of Science; n, number of patients; PhD, Doctor of Philosophy.

### Questionnaire components

The Views on Education and Nutrition in Scleroderma (VENUS) questionnaire, developed by Samm *et al,*^15^ was translated into the Greek language and distributed to rheumatologists. The VENUS questionnaire consists of 13 items, structured into three thematic domains, namely demographic information, scleroderma management and methods of providing nutrition education.

The demographics domain includes questions regarding work setting and time spent treating patients with scleroderma. We further explored age, gender, region of practice and educational level of participants. The second domain focuses on patient management and symptoms and includes four items. Initially, responders were asked to indicate how often they observed specific symptoms (arthritis, contractures, ulcers, pain, GI issues or other symptoms), using a five-point Likert scale, ranging from ‘never’ to ‘always’. Additional items regarded pain site and the nature of GI issues. Furthermore, participants were asked about the frequency of observed weight loss on a five-point Likert scale and whether most patients required assistance in cooking. An open-ended question collected information regarding the specific type of help each patient required. Finally, the last question in this domain assessed food-avoiding strategies or adherence to specific diets as a means to manage SSc symptoms.

The final domain of the VENUS Questionnaire^15^ includes five items, focusing on identifying nutrition education needs of patients with SSc. Rheumatologists were asked about the specific types of nutrition information that patients require, whether dietitian consultation would be beneficial, and what were the preferred modes of delivering dietary advice (eg, face-to-face, telephone, written resources). Additional questions regard the frequency of dietary education, the degree of confidence for student-led dietary advice for their patients and whether they would find nutrition education beneficial for themselves as medical practitioners.

### Translation of the VENUS questionnaire

With permission from the original authors,^15^ the questionnaire was translated into the Greek language using a forward-backward method by a team of three clinical dietitians and one rheumatologist, to ensure linguistic accuracy and content relevance. Minor cultural adaptations were implemented without altering the original structure of the questionnaire.

### Patient and public involvement

Patients with SSc were not involved in the study. Instead, their doctors were involved.

### Statistical analyses

Descriptive statistics were calculated for all demographic variables. For continuous variables, mean and standard deviations (SD) are reported if they are normally distributed. Otherwise, median and interquartile range (IQR) are reported. Normality of the data was assessed with the Shapiro-Wilk test. Categorical data are presented with counts (n) and percentages (%). Mann-Whitney U or χ^2^ tests were applied—wherever appropriate—to compare differences between male and female practitioners. For the purposes of the statistical analysis, only the structured, predefined questions of the questionnaire were included. Open-ended questions were not analysed quantitatively; instead, responses were reported descriptively. Associations between age and binary outcomes were examined using t-tests when normality assumptions were met. Associations between age and ordinal/nominal variables were assessed with the Kruskal-Wallis test and Spearman’s correlation, where appropriate. Statistical analyses were performed using the R Studio (V.4.3.2 (2023-10-31), R Foundation for Statistical Computing, Vienna, Austria). A *p* value<0.05 was considered as statistically significant.

## Results

### Need for nutrition education

**Figure 1:**
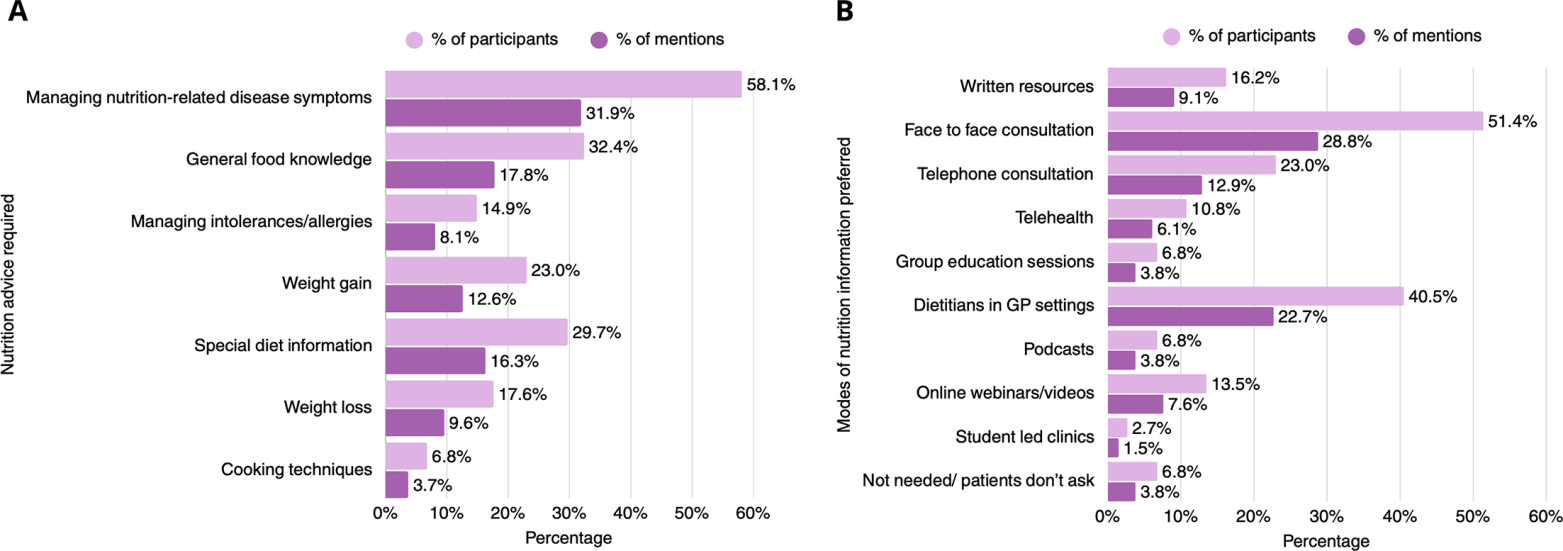
Need for nutrition education among patients with systemic sclerosis, according to the clinicians (n=74). Darker purple bars denote respondent-led percentages (participants), while lighter purple bars denote response-led percentages (mentions). GP, general practitioner.

The majority of rheumatologists recognised the necessity of patient education on matters regarding nutrition. When asked about key areas that patients require nutrition information, rheumatologists acknowledged managing nutrition-related scleroderma symptoms (58.1%), general food/nutrition knowledge (32.4%) and information on adhering to special diets (29.7%) (figure 1A). Other identified areas included the role and efficacy of micronutrient supplementation, probiotics and hypercaloric diets. Additionally, a high proportion of medical practitioners (95.9%) considered dietary consultation as beneficial for their patients. Preferred modes of dietary education included face-to-face dietitian consultation (51.4%) and dietitians in general practice settings, as part of a multidisciplinary SSc management team (40.5%) (figure 1B). Other suggested means of delivering nutrition education to the patients included information offered through Rheumatology Societies, patient support groups and multidisciplinary meetings with gastroenterologists, dietitians and rheumatologists. Most rheumatologists were hesitant on a medical undergraduate student delivering nutrition education to the patients (40.5%), while some were fairly confident (36.5%) regarding the nutritional knowledge of medical students. Half of the responders (50.0%) believed that nutrition should be discussed according to symptoms, while 17.6% preferred a nutrition-related discussion to take place at the time of SSc diagnosis. Few rheumatologists considered a monthly nutrition-themed discussion as more appropriate (13.5%), while 2.7% believed that their patients did not require face-to-face dietary consultations, but acknowledged the usefulness of online resources. An additional 2.7% of the responders did not foresee the need for nutrition education at the time of the study. Finally, 91.9% of healthcare professionals expressed eagerness in receiving continuing nutritional education, themselves.

### Gender and age-based differences

No differences were observed between male and female health professionals regarding the appropriate timing of discussing nutrition information with their patients (*p*=0.30), their confidence in student-led advice (*p*=0.74) or the frequency of observed weight loss among patients (*p*=0.13). Furthermore, no differences were observed in the frequency of patients requiring help in cooking (*p*=0.16) or advice on avoiding specific foods, according to female and male health practitioners (*p=*0.27).

A positive association was noted between age and trust in student-led dietary advice (Spearman’s rho=0.39, *p=*0.0005), suggesting that older practitioners tend to express more trust in nutrition students offering dietary advice to their patients. This is also supported by the Kruskal-Wallis test (*p*=0.014), with age distributions differing across confidence levels. Age was not associated with food avoidance (*p=*0.49) or requiring cooking assistance (*p=*0.49). In addition, no associations were observed between practitioner age and perceived patient weight loss (*p=*0.17), or regarding the appropriate timing that diet should be discussed with patients (*p=*0.25). Finally, participant practice setting was not associated with how often patients required assistance with cooking or avoided specific foods.

### Patient management and symptoms domain

**Figure 2:**
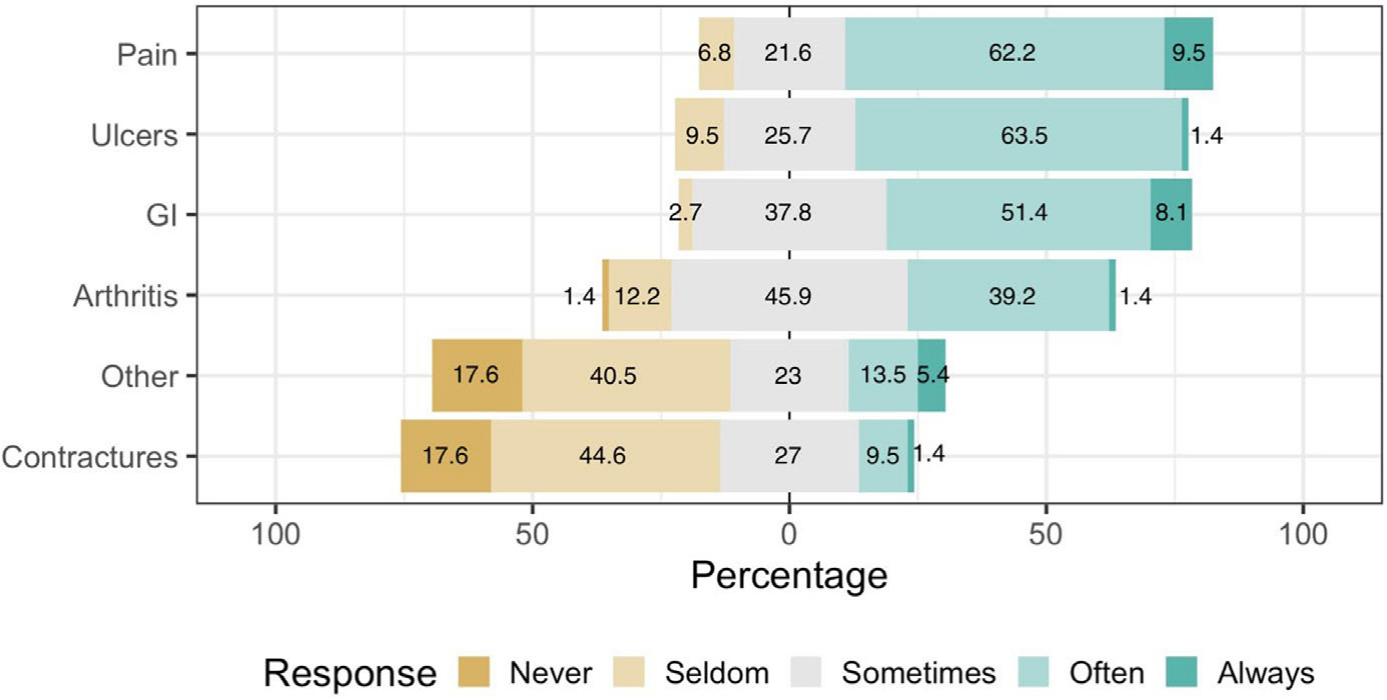
Frequency of scleroderma and related symptoms observed in clinical practice (n=74). GI, gastrointestinal.

All participants reported consulting patients with SSc-related symptoms. In further detail, 63.5% and 62.2% of the practitioners reported frequently consulting patients with ulcers and pain/soreness, respectively. Nearly half (51.4%) of the rheumatologists noted that their patients ‘often’ complained suffering from GI issues. Arthritis and contractures were less commonly encountered symptoms. Regarding arthritis, 45.9% of the responders noted that relevant symptoms are ‘sometimes’ encountered. On the other hand, according to 44.6% of the sample contractures were ‘seldom’ noted. More details can be observed in figure 2. At the open-ended question regarding the types of other symptoms, answers included dyspnoea, cough, Raynaud’s phenomenon, calcinosis, fatigue, depression and pruritus.

**Figure 3:**
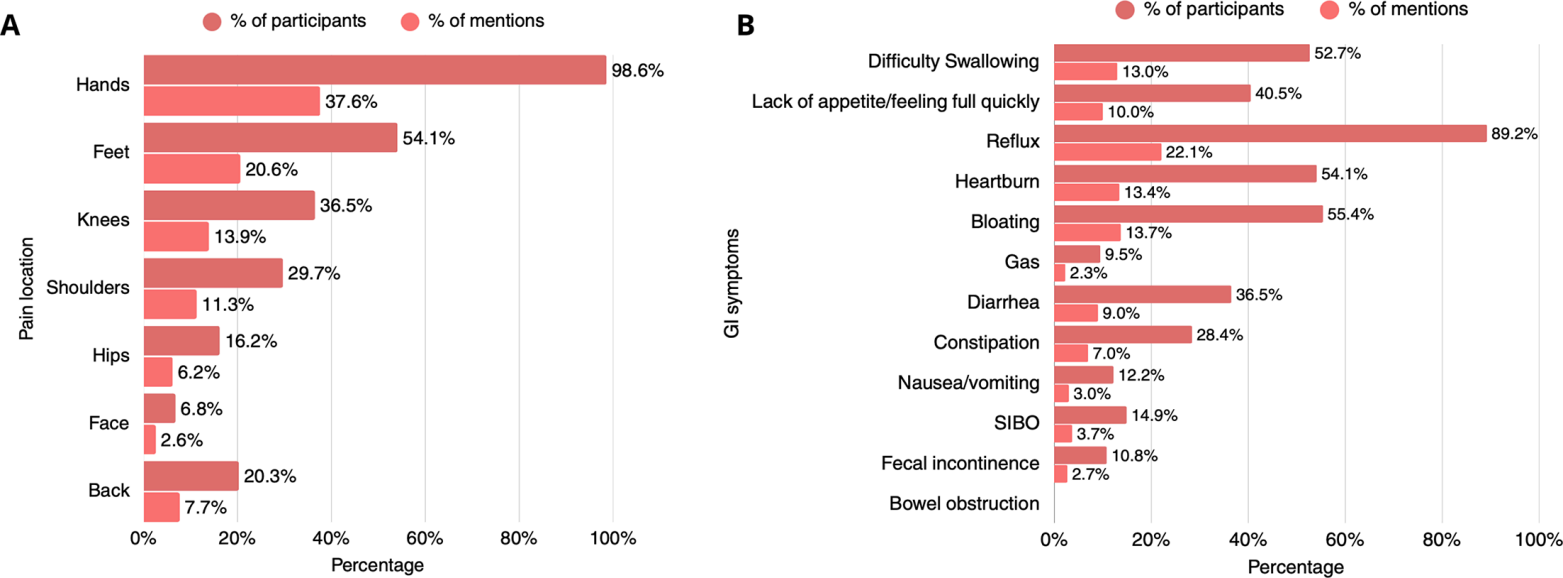
Pain locations and reported GI symptoms in patients with systemic sclerosis, as reported by the physicians (n=74). Darker red bars denote respondent-led percentages (participants), while lighter red bars denote response-led percentages (mentions). GI, gastrointestinal; SIBO, small bowel bacterial overgrowth.

The next two items in this domain involved multiresponse questions regarding pain location and specific GI symptoms. Rheumatologists were asked whether their patients commonly reported pain in specific anatomic regions, namely hands, face, feet, hips, knees, shoulders and back. The hands were the most common pain location (98.6%), followed by the feet (54.1%), knees (36.5%) and shoulders (29.7%). Hand pain accounted for 37.6% of reported pain locations. More details are presented in figure 3A,B.

**Figure 4:**
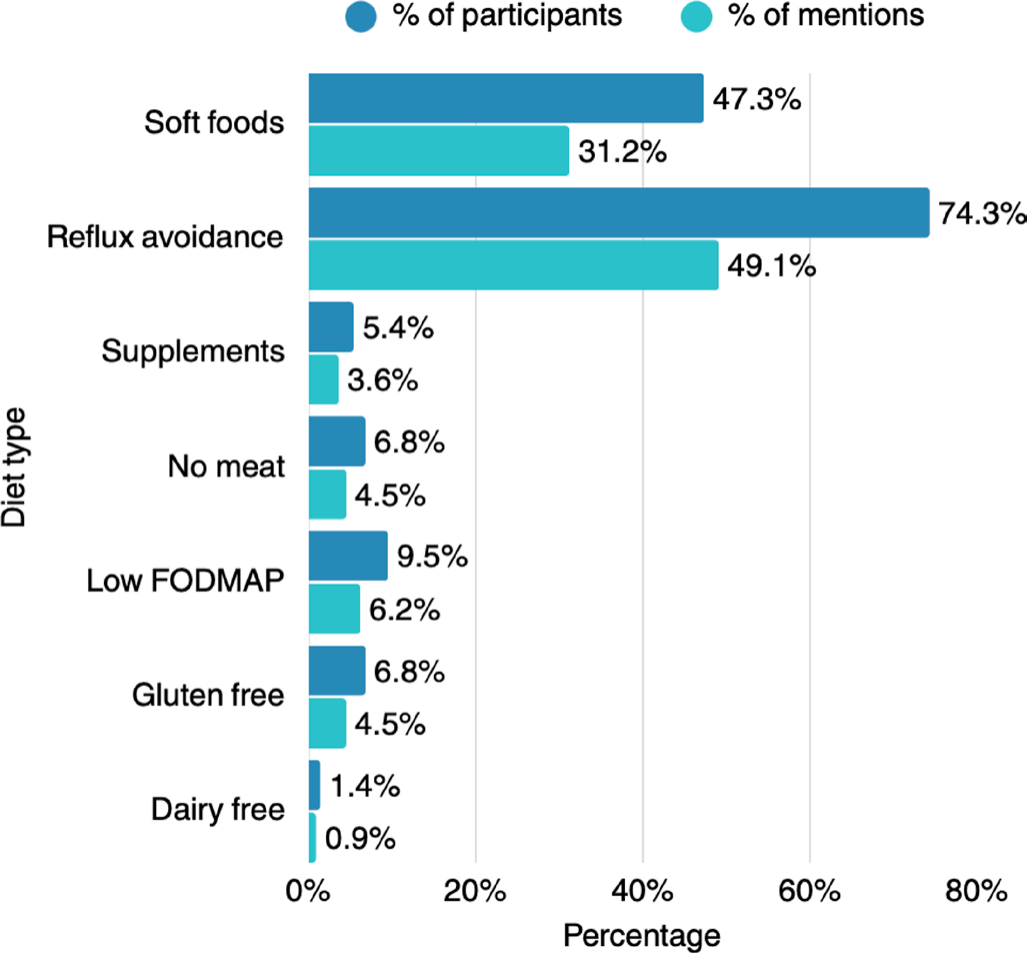
Specific dietary patterns adopted by patients with systemic sclerosis, as described by the practitioners (n=74). Darker blue bars denote respondent-led percentages (participant), while lighter blue bars denote response-led percentages (mentions). FODMAP, fermentable oligosaccharides, disaccharides, monosaccharides and polyols.

A similar multiresponse question inquired about GI symptoms, including reflux, difficulty swallowing, nausea/vomiting, lack of appetite/feeling full quickly, heartburn, constipation, gas, diarrhoea, bloating, small intestine bacterial overgrowth, bowel obstruction and faecal incontinence (figure 3B). The most frequently reported GI symptoms included GI reflux, identified by 89.2% of responders, followed by bloating (55.4%), heartburn (54.1%) and difficulty in swallowing (52.7%). Weight loss is a common issue among patients with SSc, as 51.4% of practitioners reported encountering unintentional weight loss ‘often’ and 37.8% observing weight loss among their patients ‘sometimes’ (figure 1). According to the physicians, most patients do not require help when cooking and grocery shopping (67.6%) and do not report avoiding foods or following specific diets (54.1%) in an effort to improve symptom control. Among those requiring support in cooking and grocery shopping, the most commonly reported issues involved difficulty in carrying bags and requiring assistance with fine motor skills, such as opening jars, and using cold water. Patients’ need for support when cooking and grocery shopping was attributed to hand-related impairments (contractures, digital ulcers, pain), as well as to fatigue, muscle weakness and mobility issues, as reported by the practitioners. The main dietary restrictions followed by patients involved reflux-promoting foods (74.3%) and hard to chew and swallow foods (47.3%), thus opting for a soft diet. Other dietary approaches such as low fermentable oligosaccharides, disaccharides, monosaccharides and polyols (FODMAP), gluten-free or dairy-free diets were adopted less frequently (figure 4).

## Discussion

The current study revealed that most rheumatologists acknowledge the strong value of dietary consultations, prefer dietitian-led approaches and want to receive nutritional education to improve their nutrition knowledge. According to rheumatologists, many patients with SSc require dietary support and medical advice, mainly due to hand impairments, fatigue, weakness and mobility issues. However, applied dietary modifications are often limited to reflux-avoidance and soft foods intake. The most prevalent symptoms observed in patients with SSc include ulcers, pain/soreness and GI issues. Pain sensation is predominantly presented at the upper extremities, while reflux predominates among GI symptoms.

Interestingly, most rheumatologists recognised that patients express a strong need for nutritional guidance as a complementary symptom management approach. Respondents further emphasised that patients actively seek tailored information regarding dietary modifications, and more specifically, advice related to reflux management and the selection of soft-textured foods, reflecting the impact of upper GI symptoms. Additionally, there was notable interest in structured approaches such as the low FODMAP diet, which is suggested to improve symptomatology,^16^ although the evidence remains limited.^17^

The need for registered dietitian-led support in SSc has been emphasised in the literature. Patients frequently seek information from a variety of sources, with 58% reporting engagement with healthcare professionals, 85% consulting printed media sources and 77% relying on web-based or social media platforms.^14^ In the current study, rheumatologists identified face-to-face consultations, particularly with dietitians in primary care settings, as the most effective way to deliver nutritional information to their patients. Additional approaches, including telephone consultations and written materials, were also suggested, aligning with previous reports.^15^

A salient finding is that practitioners clearly view dietary consultation as beneficial for their patients. However, despite recognising this need, the provision of nutritional guidance in SSc remains limited. Asides from limited society-issued recommendations, there is no formal consensus on the nutritional requirements in this patient group, and general practitioners often lack sufficient knowledge in this area.^16^ Additionally, patients often lack guidance on what to eat during intravenous sessions of immunotherapy, highlighting the need for nutritional support to improve therapy and disease outcomes.^18^ These discrepancies highlight the urgent need for developing structured strategies and evidence-based recommendations to support both clinicians and patients in the nutritional management of SSc. Indeed, general practitioners reported a lack of knowledge regarding clinical nutrition,^19^ which rendered them unable to provide appropriate and sufficient nutritional support. In addition, the necessity for nutrition education in medical schools has not been sufficiently met, and efforts are currently underway to incorporate nutrition-related information in the medical curriculum.^20^ Given that patients with SSc are at increased risk of developing sacropenia,^7^ dysphagia^21^ and micronutrient deficiencies^16^ and that malnutrition has been reported in 72.4% of the population,^6^ implementing early and periodic nutritional assessment may help prevent complications and improve clinical outcomes.

In SSc, the GI tract is the second most affected organ after the skin,^22^ and patients often report symptoms involving the entire tract.^23^ Consistent with prior literature, reflux and heartburn appear to be the most frequently reported complaints, with some studies estimating their prevalence at up to 80% of affected patients.^24^ Similarly, in our study, rheumatologists indicated that more than half of their patients experience reflux, heartburn, bloating and dysphagia. Notably, nearly all respondents reported encountering GI issues in their practice, to varying degrees —‘sometimes’, ‘often’ or ‘always’— underscoring how common these problems are in daily clinical practice.^25^ The presence of these persistent symptoms extends beyond physical discomfort and has important implications for patients’ QoL. Chronic reflux, bloating or dysphagia can heighten stress, disrupt daily activities and limit social interactions during meals.^26 27^ Moreover, patients often adapt their dietary habits in response to symptoms, frequently avoiding nutrient-rich foods or whole food groups, which can predispose them to inadequate nutrition or deficiencies.^28 29^ For this, having dietetic advice and SSc-specific nutrition recommendations is crucial in managing nutrition-related symptoms and attain QoL among patients.

### Limitations of the study

Although the study was designed to include rheumatologists across Greece, most completed questionnaires were collected in person, during a conference held in Thessaly. This geographic concentration may have introduced a degree of selection bias, as practitioners from other regions may have differing experiences. Another limitation involves the structure of the questionnaire, where instead of asking practitioners to provide quantitative data regarding specific symptoms, they were only asked whether such symptoms had been observed in their practice. As the questionnaire was designed to capture general clinical impressions rather than detailed quantitative measures, it has not undergone formal psychometric validation. In addition, physicians’ reporting may have led to overestimation or underestimation of certain patients’ experiences.

## Conclusion

Patients with SSc actively seek guidance on nutrition and symptom relief, underscoring the importance of comprehensive and multidisciplinary care. Medical practitioners consulting patients with SSc consistently report pain and GI manifestations as significant clinical challenges. Rheumatologists acknowledge the importance of collaborating with dietitians, emphasising how nutritional expertise can improve patient reported outcome measures.

## Supplementary material

10.1136/jsrd-2026-000005 online supplemental file 1

## Data Availability

Data are available on reasonable request.
